# Roles of phosphatidylserine exposed on the viral envelope and cell membrane in HIV-1 replication

**DOI:** 10.1186/s12964-019-0452-1

**Published:** 2019-10-21

**Authors:** Bernadette Anne Chua, Jamie Ann Ngo, Kathy Situ, Kouki Morizono

**Affiliations:** 1 Division of Hematology and Oncology, Department of Medicine, David Geffen School of Medicine, University of California, BSRB 157-01, Charles E. Young Dr. South, Los Angeles, CA 90095 USA; 2 UCLA AIDS Institute, David Geffen School of Medicine, University of California, Los Angeles, CA 90095 USA

**Keywords:** HIV-1, Phosphatidylserine, TIM family receptors, TAM, Protein S, Gas6, Scramblase, Flippases, Phagocytosis

## Abstract

Phosphatidylserine (PtdSer) is usually present only in the inner leaf of the lipid bilayers of the cell membrane, but is exposed on the outer leaf when cells are activated and/or die. Exposure of PtdSer has physiological functions. For example, the PtdSer exposed on dead cells can serve as “eat-me signals” for phagocytes to clear dead cells by phagocytosis, which prevents autoimmune reactions and inflammation. HIV-1 induces PtdSer exposure on infected and target cells and it also exposes PtdSer on its envelope. Recent studies showed that PtdSer exposed on the HIV-1 envelope and infected and target cells can facilitate or inhibit multiple steps of HIV-1 replication.

At the virus binding and entry steps, interaction of the envelope PtdSer and the host’s PtdSer-binding molecules can enhance HIV-1 infection of cells by facilitating virus attachment. At the virus budding step, HIV-1 can be trapped on the cell surface by one family of PtdSer-binding receptors, T-cell immunoglobulin mucin domain proteins (TIM)-1, 3, and 4 expressed on virus producer cells. Although this trapping can inhibit release of HIV-1, one of the HIV-1 accessory gene products, Negative Factor (Nef), can counteract virus trapping by TIM family receptors (TIMs) by inducing the internalization of these receptors. HIV-1 infection can induce exposure of PtdSer on infected cells by inducing cell death. A soluble PtdSer-binding protein in serum, protein S, bridges PtdSer exposed on HIV-1-infected cells and a receptor tyrosine kinase, Mer, expressed on macrophages and mediate phagocytic clearance of HIV-1 infected cells. HIV-1 can also induce exposure of PtdSer on target cells at the virus binding step. Binding of HIV-1 envelope proteins to its receptor (CD4) and co-receptors (CXCR4 or CCR5) elicit signals that induce PtdSer exposure on target cells by activating TMEM16F, a phospholipid scramblase. PtdSer exposed on target cells enhances HIV-1 infection by facilitating fusion between the viral envelope and target cell membrane. Because various other phospholipid channels mediating PtdSer exposure have recently been identified, it will be of interest to examine how HIV-1 actively interacts with these molecules to manipulate PtdSer exposure levels on cells and viral envelope to support its replication.

## Background

PtdSer usually resides in the inner leaf of the cell membrane [1, 2]. When a cell dies (either by apoptosis, necroptosis, or pyroptosis), PtdSer is exposed on the surface of the cell membrane [3–5]. The exposed PtdSer is recognized by PtdSer-binding proteins of either soluble proteins or cell surface receptors, which can mediate phagocytic removal of PtdSer-exposing cells by phagocytes such as macrophages [3, 6]. Viral infection, including Influenza virus and HIV-1, can induce cell death and exposure of PtdSer [7–9]. PtdSer-dependent phagocytic removal of Influenza virus-infected cells has been shown to inhibit viral replication in in vitro and in vivo settings [10–15]. Such apoptosis-dependent phagocytic removal of infected cells has been seen with HIV-1 infection [16]. However, the molecules involved in phagocytosis of HIV-1-infected cells were largely unknown since molecules mediating PtdSer-dependent phagocytosis were not fully elucidated. Recent identification of various PtdSer-binding molecules in the research field of apoptosis enabled us to study of the molecular mechanism(s) mediating phagocytic removal of HIV-1-infected cells in a PtdSer-dependent manner [17].

PtdSer is known to be exposed on various enveloped viruses, including HIV-1, and to facilitate viral replication [18]. Recent studies have demonstrated that envelope PtdSer can be involved in facilitating and inhibiting HIV-1 replication by interacting with host’s PtdSer-recognition molecules [19, 20]. In addition, recent identification of phospholipid channels, TMEM16F, which scrambles PtdSer between the inner and outer leaflet of cell membrane [21], has enabled HIV-1 researchers to explore how HIV-1 can activate TMEM16F to expose PtdSer on target cells to facilitate viral entry [22].

## Identification of molecular mechanisms mediating envelope PtdSer-dependent binding of enveloped viruses

Although PtdSer exposed on the envelope was known to support early steps of enveloped virus infection [19, 23–26], it was not known how envelope PtdSer supports viral replication and which types of molecules on target cells interact with envelope PtdSer. We identified several PtdSer-dependent virus binding and entry mechanisms while developing an HIV-1 vector that can specifically transduce desired cell types [27, 28].

Lentiviral vectors, especially HIV-1 vectors, are widely used in both clinical and research settings because they can transduce a wide variety of cells and express their transgenes for long periods of time [29]. The ability of HIV-1 vectors to transduce a wide variety of cells is conferred by the broad tropism of the envelope protein, Vesicular stomatitis virus G protein (VSV-G), which is present on the envelope of commonly used HIV-1 vectors [30, 31] instead of HIV-1 envelope protein (gp160). This process of using envelope proteins of different types of viruses instead of the cognate envelope of the virus is termed pseudotyping and is commonly used to alter the tropisms of enveloped viral vectors. The broad tropism conferred by pseudotyping with VSV-G is useful for transducing purified cells by HIV-1 vectors in vitro. However, the specific transduction of cells of interest in vivo by systemic administration would be more ideal for in vivo transduction as it reduces adverse effects of transduction of non-target cells and increases transduction of target cells [32]. Vectors that accomplish this are called targeting vectors, and we have been developing such targeting HIV-1 vectors. There are two requirements for redirecting the tropism of HIV-1 vectors to desired cell types [32, 33]. One is to eliminate the original tropisms of pseudotyping envelope proteins by abrogating the original receptor-binding regions, and the second is to confer specific binding activities to the virus by conjugating it with targeting ligands. These manipulations can destroy the entire structure and functions of the envelope protein [34]. Because the envelope protein of Sindbis virus can be manipulated without disturbing expression, structure, and function [35], we pseudotyped HIV-1 vectors with the Sindbis virus envelope protein and mutated the original receptor-binding regions of the envelope protein [36]. These mutations eliminated the original tropisms of the vectors and successfully redirected the tropisms of the vector when conjugating the vector with targeting ligands specific to desired cell types [37].

**Fig. 1:**
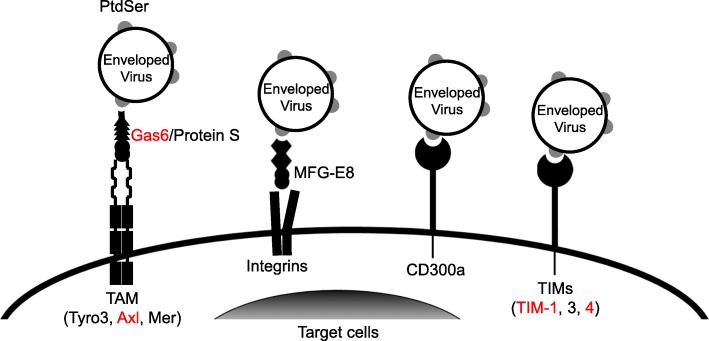
Molecular mechanisms of virus attachment mediated by envelope PtdSer. Gas6 and protein S mediate binding of the virus to target cells by bridging envelope PtdSer to TAM receptor tyrosine kinase on target cells. MFG-E8 bridges enveloped virus and target cells by binding to envelope PtdSer and to integrins αVβ3 and/or αVβ5 on target cells. TIM-1, − 3, and − 4, and CD300a are type 1 membrane proteins directly bind envelope PtdSer. Axl/Gas6 and TIM-1 and 4 (indicated in red) can mediate enveloped virus infection more efficiently than other PtdSer-binding molecules. In addition to PtdSer, TIM-1 and CD300a can mediate viral attachment by binding to phosphatidylethanolamine exposed on envelope [57, 86]

However, we found that serum contains molecules that can bridge virus to certain types of cells in an envelope protein-independent manner. Isolation and identification of a such factor(s) by FPLC and mass spectrometry revealed that the bridging molecules are soluble PtdSer-binding proteins, protein S and Gas6 [27]. Protein S and Gas6 were initially found to mediate phagocytosis of dead cells by phagocytes via bridging PtdSer exposed on dead cells to one family of receptor tyrosine kinases, TAM receptors (Tyro3, Axl, Mer), expressed on phagocytes [38, 39]. Our studies revealed that Protein S and Gas6 mediate the binding step of virus infection by binding to envelope PtdSer and TAM receptors expressed on target cells (Fig. 1). Envelope PtdSer of vaccinia virus was also known to facilitate viral replication, although the molecular mechanism(s) of how PtdSer binds to target cells was not clear [23]. We showed that the divalent binding of protein S/Gas6 to exposed envelope PtdSer and TAM receptors can facilitate vaccinia virus replication.

Because PtdSer is known to be exposed on various types of enveloped viruses, this virus binding mechanism can be used by other types of enveloped viruses. Subsequent studies of other research groups showed that protein S/Gas6 and TAM receptors can mediate PtdSer-dependent binding of wide varieties of enveloped viruses, including the Ebola, Lassa, dengue, West Nile, and Zika viruses [40–44]. Studies of our and other research groups also showed that 1) Gas6 can mediate viral infection more efficiently than protein S and 2) Axl and Tyro 3 mediate viral infection more efficiently than Mer. These are consistent with the known affinities of each of TAM receptors with either Gas6 or protein S [38].

In addition to protein S/Gas6 and TAM receptors, recent advances in cell death research identified various types of PtdSer-binding molecules that mediate phagocytic clearance of dead cells. The molecular mechanisms of PtdSer recognition are categorized in two groups based upon how they recognize PtdSer. One group is the soluble protein that bridges PtdSer on dead cells to specific receptors on phagocytes. In addition to Gas6 and protein S, MFG-E8, which binds PtdSer and integrins αVβ3/5, also belongs to this group [45, 46]. The molecules of other groups are cell surface receptors that can directly bind PtdSer, including TIM-1, 3, and 4, Stabilin 1 and 2, BAI-1, and RAGE [47–55]. To explore the possibility that these molecules can also mediate binding of enveloped viruses, we and other research groups examined all types of PtdSer receptors for their ability to mediate virus binding and entry [28, 42, 56]. These studies found that TIM-1 and -4 can efficiently mediate binding of enveloped virus (Fig. 1). Additionally, it was also found that MFG-E8, TIM-3, and CD300a can mediate virus binding, albeit less efficiently than Gas6/Axl and TIM-1 and -4 [57]. The efficiencies of PtdSer-binding molecules to mediate virus binding and entry seem to correlate with known affinities of these molecules for PtdSer [58, 59].

In addition to the viruses described above, recent studies demonstrated that Japanese encephalitis, hepatitis C, Tacaribe, and Ross River can use at least one of PtdSer-binding molecular mechanisms for their binding to cells [58–60].

## The roles of HIV-1 envelope PtdSer in virus binding and entry

These results demonstrated that PtdSer exposed on the envelope can support binding and entry of enveloped virus. Two pieces of evidence suggested that PtdSer-recognizing molecules can also mediate binding of HIV-1. First, exposed PtdSer supported HIV-1 replication of macrophages [19, 20]. Second, ectopic expression of TIM-1 on CD4+ T-cell lines was recently shown to facilitate HIV-1 entry into cells [61]. Therefore it was likely that PtdSer-binding molecules support HIV-1 entry as we observed with targeting HIV-1 vectors. However, the mechanisms by which HIV-1 mediates the fusion of the viral envelope and cell membrane are unlike the fusion mechanisms of Sindbis virus [33]. This difference could affect the role of PtdSer-binding molecules in HIV-1 entry.

**Fig. 2:**
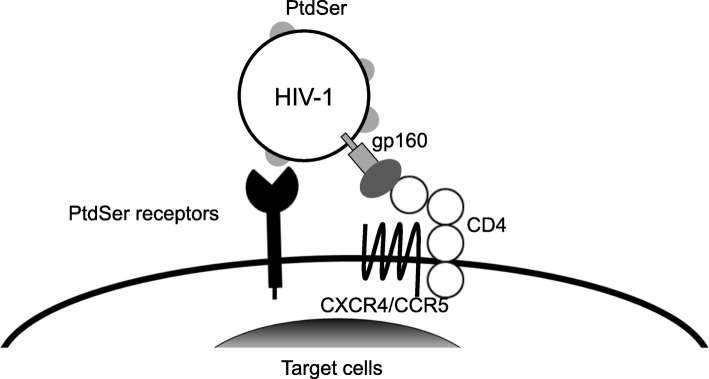
Molecular mechanism of envelope PtdSer-supported HIV-1 binding and entry. HIV-1 infection supported by envelope PtdSer. The interaction between envelope PtdSer and the host’s PtdSer-binding molecules can facilitate HIV-1 binding to target cells. The interaction of the HIV-1 envelope protein with CD4 and co-receptors (CXCR4/CCR5) is still necessary for fusion between the viral envelope and the target cell membrane

The envelope protein of Sindbis virus activates its fusion activity when exposed to a low pH environment [62]. Thus, binding via envelope PtdSer can mediate viral fusion as long as virus is endocytosed and exposed to a low pH environment. This suggests that for the envelope proteins that mediate fusion in a pH-dependent manner, PtdSer-binding molecules could serve as a viral receptor that mediates both the binding and entry steps, as long as the PtdSer can induce endocytosis of virus. In contrast, the fusion activity of HIV-1 envelope proteins, gp160, is activated by binding to their cognate receptor, CD4, and co-receptors (i.e. CXCR4 and CCR5) [63]. Thus, it is possible that the interaction between envelope PtdSer and host’s PtdSer-recognizing molecules only mediates HIV-1 binding, but not fusion steps. Since this is a critical issue for understanding the role of envelope PtdSer in the host range/tropisms of HIV-1, we examined the role of PtdSer-binding molecules in HIV-1 binding and infection in the presence and absence of the HIV-1 cognate receptor (CD4) and co-receptor (CCR5). We used a cell line, Affinofile, which changes expression levels of CD4 and CCR5 based upon culture condition [64]. We ectopically expressed TIM-1 on Affinofile cells and investigated whether expression of CD4 and/or co-receptors is necessary for HIV-1 infection when virus binding is mediated by TIM-1. Our results demonstrated that: 1) envelope PtdSer can mediate HIV-1 binding via the host’s PtdSer-binding molecule; 2) binding mediated by envelope PtdSer cannot mediate virus infection without expression of CD4 and co-receptors on target cells, most likely due to the lack of activation of fusion activity of gp160; and 3) envelope PtdSer can facilitate HIV-1 infection of CD4 and co-receptor positive cells by facilitating the virus binding step (raw data not shown but available with detailed materials and methods from the corresponding author per request) (summarized in Fig. 2). Similarly, it was previously shown that TIM-1-mediated Ebola virus infection requires expression of Ebola virus fusion receptor, Niemann-Pick C1, on target cells [58, 65].

Although PtdSer-binding molecules will not extend the host range of HIV-1 beyond CD4 and co-receptor positive cells, our data showed that these molecules can facilitate HIV-1 replication of cells expressing CD4 and one of the co-receptors. We attempted to examine the effects of PtdSer-mediated binding on HIV-1 replication in primary CD4 and co-receptor positive cells; however, we could not detect expression of the high-affinity PtdSer-binding molecules, including Axl, Tyro3, TIM-1 and 4, on CD4-positive T-cells (Th0, Th1, Th2, Th17, and Treg), macrophages (M0 and polarized to M1 and M2), and dendritic cells (DC) (please see Fig. 1d of Ref [17] and data not shown). Therefore, we have not been able to confirm the role of envelope PtdSer in HIV-1 replication in human primary immune cells. However, these primary cells are generated by in vitro differentiation and/or activation, which might not represent the phenotypes and functions of various types of T-cells, macrophages, and DC present in vivo. Identification of CD4-positive cells expressing Axl, Tyro3, or TIM-1 or 4 in vivo and isolation of such cell types will further elucidate the roles of envelope PtdSer in HIV-1 replication of primary cells.

Axl is expressed on human primary endothelial cells [27]. TIM-1 is expressed on human epithelial cells and mediated HIV-1 binding to the cells [66]. These cells do not express CD4 and therefore cannot be infected by HIV-1 according to our data summarized in Fig. 2. However, they are still able to trap HIV-1 on their surfaces. DC can efficiently mediate trans-infection of neighboring CD4-positive T-cells by trapping HIV-1 on their cell surface using DC-SIGN, which binds N-glycan of Gp160, or CD169, which binds glycosphingolipid of envelope [67–69]. It will be of interest to determine whether HIV-1 trapped on CD4-negative cells via the interaction between envelope PtdSer and PtdSer-binding molecules can be transferred to neighboring CD4-positive cells and mediate trans-infection.

## The roles of PtdSer-binding molecules in budding of HIV-1

**Fig. 3:**
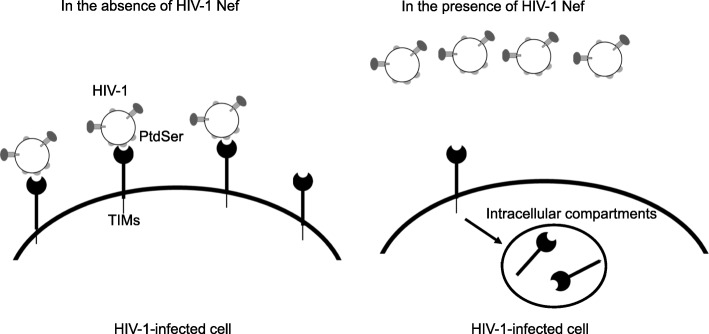
Inhibition of HIV-1 release by TIMs. TIM-1, 3, and 4 trap HIV-1 on the surface of the infected cells and inhibit release of HIV-1. The HIV-1 Nef protein antagonizes the inhibitory effects of TIMs on viral release by inducing internalization of TIMs and reducing transcription of TIMs

Envelope PtdSer can mediate binding to cells, not only at the initial virus-cell binding step, but also at the budding step. TIM-1, 3, and 4 expressed on virus producer cells were shown to bind envelope PtdSer and trap virus on virus producer cells [61]. Trapping of HIV-1 by TIMs reduces the amount of HIV-1 released in the culture supernatant (Fig. 3). Thus, PtdSer-binding mechanisms seem to function as the host’s antiviral machinery at the budding step. The expression and functions of TIMs are supported by host’s SERINC proteins [70]. The HIV-1 Nef protein, a product of one of the HIV-1 accessory genes, can reduce inhibitory effects of TIMs on virus budding by antagonizing SERINC proteins. The relationship between TIMs and Nef seems to be similar to other known antiviral molecules, such as Tetherin, which traps HIV-1 on producer cells, and Vpu, a product of one of the HIV-1 accessory genes, which inhibits the antiviral functions of Tetherin [71].

It is not known whether HIV-1 trapped on the surface of virus producer cells in a PtdSer-dependent manner is infectious. If the trapped HIV-1 is infectious, the trapped virus could efficiently infect neighboring cells by taking advantage of the cell-to-cell contact between virus producer cells and neighboring CD4-positive cells. If cell-to-cell infection can occur, HIV-1 might expose PtdSer on the envelope in order to facilitate replication via cell-to-cell infection [72].

## PtdSer-dependent phagocytosis of HIV-1-infected cells by macrophages

**Fig. 4:**
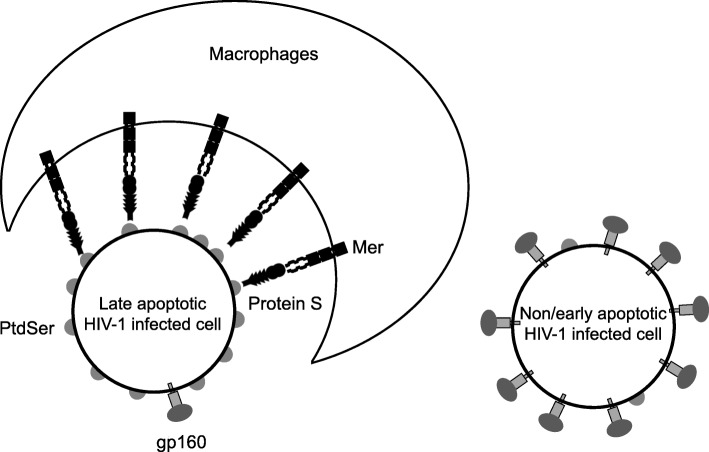
Molecular mechanism of phagocytic clearance of HIV-1-infected cells by macrophages. HIV-1 induces PtdSer exposure on CD4+ T-cells. The infected cells exposing high levels of PtdSer are at a late apoptotic stage and produce relatively low amounts of viral proteins. The cells producing viral proteins at high levels expose PtdSer at low levels. Protein S mediates phagocytic clearance of HIV-1-infected cells at a late apoptotic stage by bridging PtdSer exposed on the infected cells to Mer expressed on macrophages

It has been known that HIV-1-infected cells are phagocytosed by macrophages in vivo by a mechanism(s) independent of antiviral antibodies [16]; however, how macrophages recognize and engulf HIV-1-infected cells is not known. Because HIV-1 infection is known to induce PtdSer on infected cells, we investigated whether PtdSer and its binding molecules are involved in phagocytosis of HIV-1-infected cells [17]. We found that human serum contains a soluble molecule(s) that induces phagocytosis of HIV-1-infected cells by human primary macrophages. Concealing PtdSer exposed on HIV-1-infected cells abrogated this phagocytosis mediated by serum. More specifically, we found that phagocytosis is mediated by protein S present in serum, PtdSer exposed on HIV-1-infected cells, and Mer expressed on macrophages (Fig. 4). It is known that PtdSer-dependent phagocytosis of influenza virus-infected cells inhibits virus replication by depleting virus producer cells [10–15]. We investigated whether phagocytosis of HIV-1-infected cells can similarly inhibit virus production, and found that phagocytic removal of HIV-1-infected cells does not significantly inhibit virus production, since this phagocytic mechanism selectively removes late apoptotic cells that express high levels of PtdSer but low levels of viral proteins. This could be due to the low affinity of Mer for protein S and Gas6, which will require high levels of PtdSer exposure to mediate phagocytosis because the affinities of Mer for protein S and Gas6 are not as high as those of Axl [27, 38, 73]. If there is a phagocyte population expressing Axl, TIM-1, and/or TIM-4 in vivo, these phagocytes might be able to recognize phagocytes and early apoptotic cells that expose relatively low levels of PtdSer while producing high levels of viral proteins.

Of note, when we examined the role of MerTK in PtdSer-dependent virus entry, MerTK could not efficiently mediate viral entry [27]. It is possible that late apoptotic cells expose PtdSer at a higher density than viral envelope does. Development of a method that can stringently quantify the amount of exposed PtdSer on each virion will enable us to examine this hypothesis.

It was previously shown that PtdSer-mediated phagocytosis of LCMV-infected cells facilitates presentation of virus-derived peptides on MHC class I molecules and stimulate antiviral T-cells [74]. Thus, it is possible that phagocytosis of HIV-1 infected cells at late apoptotic stage still inhibit HIV-1 replication by raising antiviral immunity.

Baxter et.al. also showed that macrophages can bind and recognize HIV-1-infected cells in an apoptosis-dependent but an HIV-1 envelope protein-independent manner [75]. Although we only focused on phagocytic removal of HIV-1-infected cells in a PtdSer-dependent manner, their results showed that HIV-1 can exploit this binding to facilitate cell-to-cell infection of macrophages. Thus, it is also possible that expression of high-affinity PtdSer binding molecules can facilitate HIV-1 infection of macrophages by mediating cell-to-cell binding between macrophages and cells producing HIV-1 at high levels.

## Exposure of PtdSer on the cell membrane by binding of HIV-1 to target cells

Binding of alphaherpesviruses to target cells are known to induce exposure of PtdSer on target cells [76]. This PtdSer is known to facilitate viral replication. The detailed molecular mechanisms of how PtdSer is moved to the outer layer of target cell membrane after virus binding was not fully elucidated because bona fide phospholipid channels were not identified until recently [77].

Phospholipid channels that mediate symmetric and asymmetric distribution of PtdSer are categorized in two types [78]. One is a scramblase that can transport phospholipids from the inner to outer and outer to inner leaf of the cells membrane bidirectionally, disturbing the asymmetric distribution of PtdSer. The other is flippases that transport PtdSer from the outer leaf to inner leaf, which maintains an asymmetric distribution of PtdSer. Activation of scramblase, as well as inactivation of flippases, can lead to exposure of PtdSer.

**Fig. 5:**
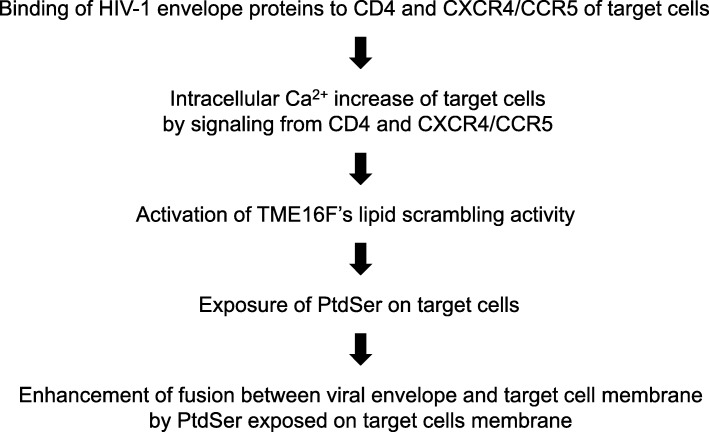
Enhancement of the viral fusion step by HIV-1-induced PtdSer exposure on target cell membranes

Dr. Nagata’s group identified two groups of scramblases. One is the scramblases activated by increase of intracellular calcium concentration (TMEM16C, D, F, G, and J) [21]. The other group of scramblases are activated by proteolysis by caspase 3 or 7 (Xkr4, 8, and 9) [79]. They also identified one group of flippases (ATP8A2 and ATP11A and C) [80]. The activities of these flippases are inhibited by both an increase of intracellular calcium concentrations and proteolysis by caspase 3. It is likely that the calcium-dependent phospholipid channels mediate activation-induced PtdSer exposure while caspase-dependent ones mediate cell death-induced PtdSer exposure. Identification of these molecules allowed the molecular mechanisms of virus-induced PtdSer exposure to be elucidated. HIV-1 was recently shown to induce PtdSer by activating scramblase [22]. This study revealed that HIV-1 binding to target cells can activate TMEM16F by eliciting signaling through CD4 and co-receptors (Fig. 5). This activation induces exposure of PtdSer on the cell membrane of target cells, and exposed PtdSer can facilitate fusion between the viral envelope and target cell membrane (note that the activation of the envelope protein via binding to CD4 and co-receptors is still required for the fusion step) [22]. This study showed that HIV-1 manipulate PtdSer exposure levels to facilitate its replication.

## The mechanisms by which HIV-1 exposes PtdSer on its envelope

Because binding of PtdSer-binding molecules to PtdSer is affected by the concentrations of exposed PtdSer on the membrane, the roles of PtdSer-binding molecules in HIV-1 replication could vary, based upon the levels of PtdSer exposure on individual virions. However, it is not known whether each virion exposes the same amount of PtdSer. Flow cytometric analysis of individual HIV-1 virions (flow virometry) can help demonstrate whether PtdSer is uniformly exposed on HIV-1 [81–83].

The means by which HIV-1 exposes PtdSer on its membrane is not well understood and could be due to simple induction of apoptosis, subsequent exposure of PtdSer on virus producer cells, and budding from the cell membrane of apoptotic virus producer cells. Our study showed that the levels of PtdSer on HIV-1-infected cells varies, depending on whether the cells are in early or late apoptotic phases; therefore, the concentrations of exposed PtdSer on HIV-1 might vary, depending on the apoptotic phases of virus producer cells [17].

Ebola virus is known to incorporate activated Xkr8 in virus, which induces exposure of PtdSer on the envelope [84]. Another recent study demonstrated that Ebola virus activates TMEM16F of infected cells, resulting in PtdSer exposure on virus producer cells and the envelope [85]. It will be of interest to determine whether HIV-1 can also interact with scramblases and/or flippases to change the levels of PtdSer exposure in viral envelope and viral producer cells.

Of note, we have observed that the effects of PtdSer binding molecules (Axl/Gas6 and TIM-1) on lentiviral titers differ among various pseudotypes [28]. It is possible that different types of envelope proteins can induce PtdSer exposure on virus and/or viral vectors to different extents, which can be caused by differences in their interactions with scramblases and/or flippases. Quantitative analysis of PtdSer exposure on each virion of lentiviral vectors will be necessary to elucidate the molecular mechanisms of this phenomenon.

## Conclusion

This review highlights the different roles of exposed PtdSer in HIV-1 replication. PtdSer exposed on HIV-1-infected cells mediates phagocytic removal of the infected cells, and PtdSer exposed on target cells facilitates the HIV-1 fusion step. PtdSer exposed on the HIV-1 envelope can facilitate viral binding while inhibiting viral release via interactions with PtdSer-binding molecules.

Because these roles can either facilitate or inhibit viral replication at different stages of virus life cycles, it is important to set up appropriate experimental settings to study exposed PtdSer in a particular viral replication step of interest [61, 70].

The above-mentioned roles of envelope PtdSer in viral replication were clarified through advances in the understanding of the molecular mechanisms of recognition of apoptotic cells and PtdSer exposure. Recent identification of molecules of PtdSer exposure machinery will enable further investigation of the abilities of viruses to control PtdSer exposure levels on their envelopes and virus producer cells in order to support their replication, which could lead to new antiviral strategies targeting virus-induced exposure of PtdSer.

## Data Availability

The datasets used and/or analyzed during the current study are available from the corresponding authors on reasonable request.

## Abbreviations

DC: Dendritic cell
Gas6: Growth Arrest Specific 6
HIV-1: Human Immunodeficiency Virus Type 1
LCMV: Lymphocytic choriomeningitis virus
MFG-E8: Milk Fat Globule-EGF Factor 8 Protein
Nef: Negative Factor
PtdSer: phosphatidylserine
TIM: T-cell immunoglobulin mucin domain proteins
TIMs: TIM family receptors
VSV-G: Vesicular stomatitis virus G protein
